# Cardiopulmonary examinations of athletes returning to high-intensity sport activity following SARS-CoV-2 infection

**DOI:** 10.1038/s41598-022-24486-x

**Published:** 2022-12-15

**Authors:** Mate Babity, Mark Zamodics, Albert Konig, Anna Reka Kiss, Marton Horvath, Zsofia Gregor, Reka Rakoczi, Eva Kovacs, Alexandra Fabian, Marton Tokodi, Nora Sydo, Emese Csulak, Vencel Juhasz, Balint Karoly Lakatos, Hajnalka Vago, Attila Kovacs, Bela Merkely, Orsolya Kiss

**Affiliations:** 1 Heart and Vascular Center, Semmelweis University, 68 Varosmajor Street, Budapest, 1122 Hungary; 2 Department of Sports Medicine, Semmelweis University, 68 Varosmajor Street, Budapest, 1122 Hungary

**Keywords:** Cardiology, Diseases, Medical research

## Abstract

After SARS-CoV-2 infection, strict recommendations for return-to-sport were published. However, data are insufficient about the long-term effects on athletic performance. After suffering SARS-CoV-2 infection, and returning to maximal-intensity trainings, control examinations were performed with vita-maxima cardiopulmonary exercise testing (CPET). From various sports, 165 asymptomatic elite athletes (male: 122, age: 20y (IQR: 17-24y), training:16 h/w (IQR: 12–20 h/w), follow-up:93.5 days (IQR: 66.8–130.0 days) were examined. During CPET examinations, athletes achieved 94.7 ± 4.3% of maximal heart rate, 50.9 ± 6.0 mL/kg/min maximal oxygen uptake (V̇O_2max_), and 143.7 ± 30.4L/min maximal ventilation. Exercise induced arrhythmias (n = 7), significant horizontal/descending ST-depression (n = 3), ischemic heart disease (n = 1), hypertension (n = 7), slightly elevated pulmonary pressure (n = 2), and training-related hs-Troponin-T increase (n = 1) were revealed. Self-controlled CPET comparisons were performed in 62 athletes: due to intensive re-building training, exercise time, V̇O_2max_ and ventilation increased compared to pre-COVID-19 results. However, exercise capacity decreased in 6 athletes. Further 18 athletes with ongoing minor long post-COVID symptoms, pathological ECG (ischemic ST-T changes, and arrhythmias) or laboratory findings (hsTroponin-T elevation) were controlled. Previous SARS-CoV-2-related myocarditis (n = 1), ischaemic heart disease (n = 1), anomalous coronary artery origin (n = 1), significant ventricular (n = 2) or atrial (n = 1) arrhythmias were diagnosed. Three months after SARS-CoV-2 infection, most of the athletes had satisfactory fitness levels. Some cases with SARS-CoV-2 related or not related pathologies requiring further examinations, treatment, or follow-up were revealed.

## Introduction

Severe acute respiratory syndrome coronavirus 2 (SARS-CoV-2) is responsible for coronavirus disease 2019 (COVID-19), which can cause cardiac complications besides pulmonary and various other diseases^1^. During the SARS-CoV-2 pandemic, multiple studies were carried out on the short-term effects of acute SARS-CoV-2 infection in athletes^2–4^. In most studies, athletes had mild-to-moderate symptoms of COVID-19 during the acute phase of the infection, while some cases of young athletes with serious complications—including myocarditis, thromboembolism, severe pneumonia and even sudden death—were reported^5,6^.

Initial data suggested a large proportion of myocarditis among patients due to SARS-CoV-2 infection, however further studies described considerably less cases^7–9^. During the first wave of the pandemic, only viewpoints and expert comments helped us in the evaluation of athletes after COVID-19, while increasing amount of data about the infection fostered strict recommendations for return-to-sport^10–15^. Due to these recommendations, return-to-sport cardiology examination protocol included resting electrocardiogram (ECG), cardiac troponin level measurement and echocardiographic examinations^16,17^. Later, some literature data indicated the occurrence of severe cardiac involvement was rare. Thus, detailed examinations may be unnecessary for athletes who were asymptomatic or mild-symptomatic, but without cardiac symptoms, during the infection^18^.

However, previously hospitalized patients who had moderate-to-severe COVID-19, negative effects on multiple vital organs and on fitness status could be revealed even 2–3 months after the infection^19^. In athletes, long-term symptoms like fatigue or subjective feeling of decreased training capacity were also reported after SARS-CoV-2 infection^20^. Plenty of data are available in the literature about the cardiopulmonary status of athletes right after the infection, while we have less information about athletes who have already returned to high-intensity training after recovering from COVID-19 infection. In a longitudinal view for athletes, the long term alterations in physical fitness as well as the probable time interval until reachieving the peak-performance could be more important after SARS-CoV-2 infections. With these information the athletes and their coaches would be able to perform better training-planning.

Our aims were to evaluate residual symptoms and exercise capacity of athletes after returning to maximal intensity training following SARS-CoV-2 infection, and to compare the cardiopulmonary exercise testing (CPET) results performed before and after SARS-CoV-2 infection to detect the possible negative long-term effects of the infection.

## Methods

In a one-year period from 2020 autumn, cardiology control examinations and CPET were carried out following returning to high intensity trainings after suffering a SARS-CoV-2 infection in 183 athletes. Asymptomatic elite athletes (adults training ≥ 10 h/week), and all athletes with positive findings or ongoing symptoms during the first cardiology check-up were invited to take part in the study. Athletes with previously known cardiovascular diseases (excluding hypertension), or with musculoskeletal symptoms were excluded from the study.

Detailed CPET analysis was carried out in 165 asymptomatic elite athletes. Moreover, the results of cardiovascular evaluation of 18 athletes either symptomatic or with previous pathological findings were examined separately and individually.

Prior to the study, all participants gave written informed consent to the examinations, and the Medical Research Council of Hungary approved the study (No.: IV/9697-1/2020/EKU) according to the Ethical Guidelines of the Helsinki Declaration and to Good Clinical Practice. All measurements were performed at least 12 h after the last training session^21^.

The SARS-CoV-2 infection was confirmed by polymerase chain reaction (PCR) or by rapid antigen test (RAT), these tests were carried out individually prior the study and were necessary for the enrolment. Athletes underwent cardiology screening in accordance with the return-to-sport recommendations 2–3 weeks after the infection^16,17^. After the first screenings the athletes were advised to build up their regular training step-by-step. Athletes were invited for the second examinations between 2 and 4 months after the SARS-CoV-2 infection, immediately after they have already returned to their current maximal intensity training. The current cross-sectional and self-controlled study analyses the results of these second cardiology screening measurements containing CPET examinations of the athletes.

A detailed questionnaire was implemented to record the data of SARS-CoV-2 infection and sport activity. The severity of the acute infection was classified in accordance with the recommendations of Löllgen H et al.^22^ Following physical examination and blood pressure measurement (Omron M6 Comfort, OMRON Healthcare Group, Japan), 12-lead ECG (CardioSoft PC, GE Healthcare, Finland) was recorded in a resting, laying position and analysed according to the current guidelines^23–25^. Cardiac necroenzyme levels were measured from blood samples (cobas e 411 analyzer, ROCHE Hungary Ltd, Hungary; Elecsys Troponin T hs, Roche Diagnostics International AG, Switzerland^26^). Control echocardiography was performed according to the current guidelines (Vivid E95, GE Vingmed Ultrasound, Norway)^27^.

Maximal CPET was carried out on a treadmill (T-2100, GE Healthcare, Finland) with sport-specific incremental protocols (starting with a 1-min sitting resting phase, followed by 1–2 min flat walk of 6 km/h as warm-up, then by continuous 8–10 km/h uphill running with an increasing slope of 1.0–1,5% every minute until exhaustion). Maximal intensity was considered to be achieved, if the athlete reported maximal subjective exhaustion and either the respiratory exchange ratio (RER) was over 1.1, and/or flattening could be seen in the oxygen uptake and the heart rate curves. After stopping running, measurements were continued during a 1-min 4 km/h walk and a further 4-min rest^28,29^. Breath-by-breath gas analysis was carried out with an automated cardiopulmonary exercise system (Respiratory Ergostik, Geratherm, Germany). The reference values for non-athletes were integrated to the system by the manufacturer considering sex, age, height, and weight. During the CPET examinations, continuous ECG monitorization was carried out (CAM-14 module, GE Healthcare, Finland), the estimated maximal heart rate was calculated as *220-age*^30^. Blood lactate levels were measured at rest, every second minute during the exercise, at maximal load and in the fifth minute of the recovery phase (Laktate Scout 4+ , EKF Diagnostik, Germany). Anaerobic threshold was determined considering lactate curves and the kinetics of the recorded Wasserman graphs^31^. All CPET data were reported as an average of 10 s. If previous examinations indicated, further examinations were also carried out (24-h Holter ECG, 24-h ambulatory blood pressure measurements, cardiac CT, cardiac MRI, stress echocardiography, cardiac percutaneous coronary intervention). In cases of athletes with previous CPET data from before the SARS-CoV-2 infection, the same CPET protocols were applied for both examinations and comparisons were made between pre- and post-SARS-CoV-2 CPET measurements. The pre-SARS-CoV-2 CPET examinations took place in different training status (off season, preparation period, peak performance), while the post-SARS-CoV2 CPET examinations were carried out after a period of 2–3 weeks of training break during SARS-CoV-2 infection followed by a step-by-step re-building training to reachieve peak performance. For inclusion to the study it was mandatory to achieve maximal intensity at the CPET examination after the infection, and, in case of CPET comparisons, in the CPET examination before the infection as well. All examinations and data collection were supervised by a cardiology and sports medicine specialist.

Statistical analyses were performed using a dedicated software (Microsoft Excel, Microsoft Corporation, USA; Real Statistics Resource Pack software (Release 7.6), Copyright (2013–2021) Charles Zaiontz)^32^. Descriptive statistical values are shown as number (percentage), as mean ± SD for normally distributed parameters, as median (interquartile range: 1st quartile –3rd quartile (IQR: Q1–Q3)) for non-normally distributed parameters. Shapiro–Wilk Test was performed for testing the normality of the parameters. Comprehensive statistical analysis was carried out with paired Student’s t-test or Wilcoxon Signed Rank Test, depending on the normality of the data. Statistical significance was determined if *p* < 0.05. All missing data were proved to be missing-completely-random, thereby available-case-analysis was carried out for the statistical evaluation. The data underlying this article will be shared on reasonable request to the corresponding author.

## Results

Cardiology control measurements after returning to high intensity trainings following SARS-CoV-2 infection were performed in 165 asymptomatic elite athletes and 18 symptomatic athletes or athletes with pathological findings.

### Results of asymptomatic elite athletes

**Figure 1:**
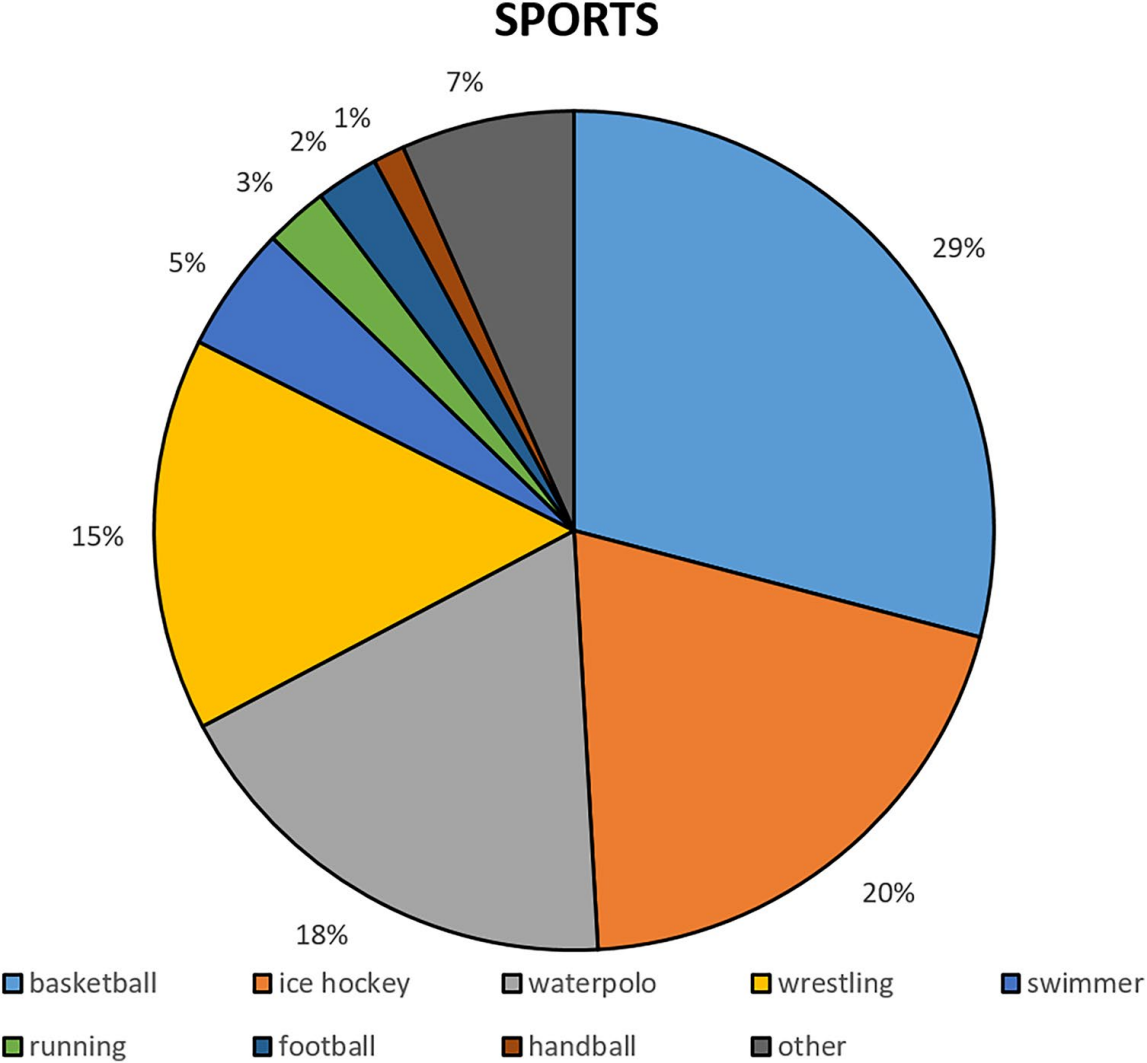
Types of sports of the examined asymptomatic elite athletes.

The analysis was performed in 165 asymptomatic elite athletes (male: 122 (73.9%), age: 20 years (IQR: 17–24 years), training: 16 h/week (IQR: 12–20 h/week)) from various types of sport (Fig. 1), 93.5 days (IQR: 66.8–130.0 days) after the first signs of the infection and after 21 days (IQR: 14–28 days) of training cessation.

During the acute phase, 28 (17.0%) athletes had asymptomatic infection, 136 (82.4%) had mild symptoms, while 1 (0.6%) athletes had moderate symptoms due to the SARS-CoV-2 infection.

Slightly elevated high-sensitive Troponin T (hs Troponin T) levels were measured in one (0.6%) elite asymptomatic athlete. In this case, slightly elevated hs Troponin T level was present 4 months after the SARS-CoV-2 infection and all other laboratory blood measurements, echocardiography and CPET examinations were negative. After skipping trainings for two weeks, hs Troponin T level normalized according to the control laboratory measurements. Therefore, the hs Troponin change was considered as sports related in this case.

Control echocardiographic examinations proved slightly increased pulmonary pressure in two (1.2%) asymptomatic elite athletes (32 + 5 mmHg and 36 + 3 mmHg), no other supposedly COVID-19-related changes were measured. In these cases, chest x-ray examinations were carried out without any pathological results. Further controls performed 7–14 days later showed normal pulmonary pressure values and no additional abnormalities were recognized. Furthermore, independently from SARS-CoV-2 infection, at the echocardiographic examinations preserved left and right ventricular ejection fraction (n = 1, 0.6%), slight diastolic dysfunction (n = 1, 0.6%), Barlow type mitral valve with mitral annular disjunction (n = 1, 0.6%) and left ventricular hypertrabecularization (n = 2, 1.2%) were revealed.

Most of the asymptomatic elite athletes had satisfactory fitness levels as per the results of CPET. (Table 1.) Resting heart rate was 70 BPM (IQR: 64–79 BPM). During CPET examinations, the athletes achieved a maximum heart rate of 187 BPM (IQR: 181–194.5 BPM) (94.7 ± 4.3% of the calculated maximal heart rate), a maximal relative aerobic power (oxygen uptake, V̇O_2max_) of 50.9 ± 6.0 ml/kg/min, and a maximal ventilation of 143.7 ± 30.4 l/min. The athletes reached their anaerobic threshold at 87.0 ± 6.4% of their V̇O_2max_, with a heart rate of 93.2% (IQR: 90.7–95.3%) of their maximal values. The 1-min heart rate recovery was 27 BPM (IQR: 22–34 BPM).

**Table 1:** CPET follow-up results of elite asymptomatic athletes after a SARS-CoV-2 infection.

	CPET results after SARS-CoV-2 (N = 165)	CPET follow-up results (N = 62; follow-up time: 0.7 years (IQR: 0.6–1.8 years))		
		Before SARS-CoV-2	After SARS-CoV-2	*p* (between before and after SARS-CoV-2)
HR_resting_ (BPM)	70 (64–79)	71.5 ± 14.3	69.3 ± 13.6	0.174
HR_max_ (BPM)	187 (181–194.5)	190.0 (183.3–200.0)	187.0 (181.0–196.0)	0.024
HR_max_ (% of calculated max.)	94.7 ± 4.3	95.6 ± 5.0	94.3 ± 4.4	0.077
HR at the anaerobic threshold (BPM)	173.0 (166.0–184.0)	170.2 (163.0–179.0)	171.1 (166.0–179.3)	0.277
HR at the anaerobic threshold (% of HR_max_)	93.2 (90.7–95.3)	90.6 (86.3–93.5)	91.4 (90.2–93.4)	0.004
HR_recovery_ (BPM)	27 (22–34)	29.0 (22.3–34.8)	27 (21.0–34.3)	0.290
VO_2max_ (ml/kg/min)	50.9 ± 6.0	49.9 ± 5.6	52.2 ± 5.6	0.004
VO_2_ at the anaerobic threshold (ml/kg/min)	44.2 ± 5.5	41.8 ± 4.5	44.2 ± 5.0	< 0.001
VO_2_ at the anaerobic threshold (% of VO_2max_)	87.0 ± 6.4	84.0 ± 7.4	85.1 ± 7.3	0.289
Ventilation (l/min)	143.7 ± 30.4	146.9 ± 27.8	155.3 ± 29.2	0.008
Maximal lactate (mmol/l)	8.1 (6.6–10.0)	8.5 (7.0–11.1)	8.7 (6.8–10.6)	0.465
Exercise time (min)	13.1 (11.0–15.0)	13.0 (11.0–15.0)	14.0 (12.0–15.8)	0.003
Anaerobic time (min)	9.2 ± 2.6	8.5 ± 2.8	9.5 ± 2.8	0.003
Anaerobic time (% of exercise time)	69.0 (61.2–77.3)	62.7 ± 14.3	66.9 ± 13.6	0.113
FEV1 (% of the expected value)	100.8 ± 16.8	105.3 ± 14.7	107.1 ± 17.6	0.198
VE/VCO_2_ slope	26.3 ± 3.0	26.2 (24.3–28.3)	25.7 (24.7–27.7)	0.713

All CPET following SARS-CoV-2 infection (N = 165) and for comprehension the results before and after SARS-CoV-2 infection (N = 62). Follow-up time after the onset of the first symptoms of SARS-CoV-2 infection: 93.5 days (IQR: 66.8–130.0 days). Time between the CPET examinations before and after SARS-CoV-2 infection: 0.7 years (IQR: 0.6–1.8 years). *CPET* Cardiopulmonary exercise testing; *SARS-CoV-2* Severe acute respiratory syndrome coronavirus 2; *HR*_*resting*_ Resting heart rate; *BPM* Beats per minute; *HR*_*max*_ Maximal heart rate; *HR*_*recovery*_ First minute heart-rate recovery after exercise testing; *VO*_*2max*_ Maximal oxygen uptake; *VO*_*2*_ Oxygen uptake; *VE/VCO*_*2*_ Slope, ventilatory efficiency slope.

### Comparison of CPET results before and after a SARS-CoV-2 infection in elite athletes

**Figure 2:**
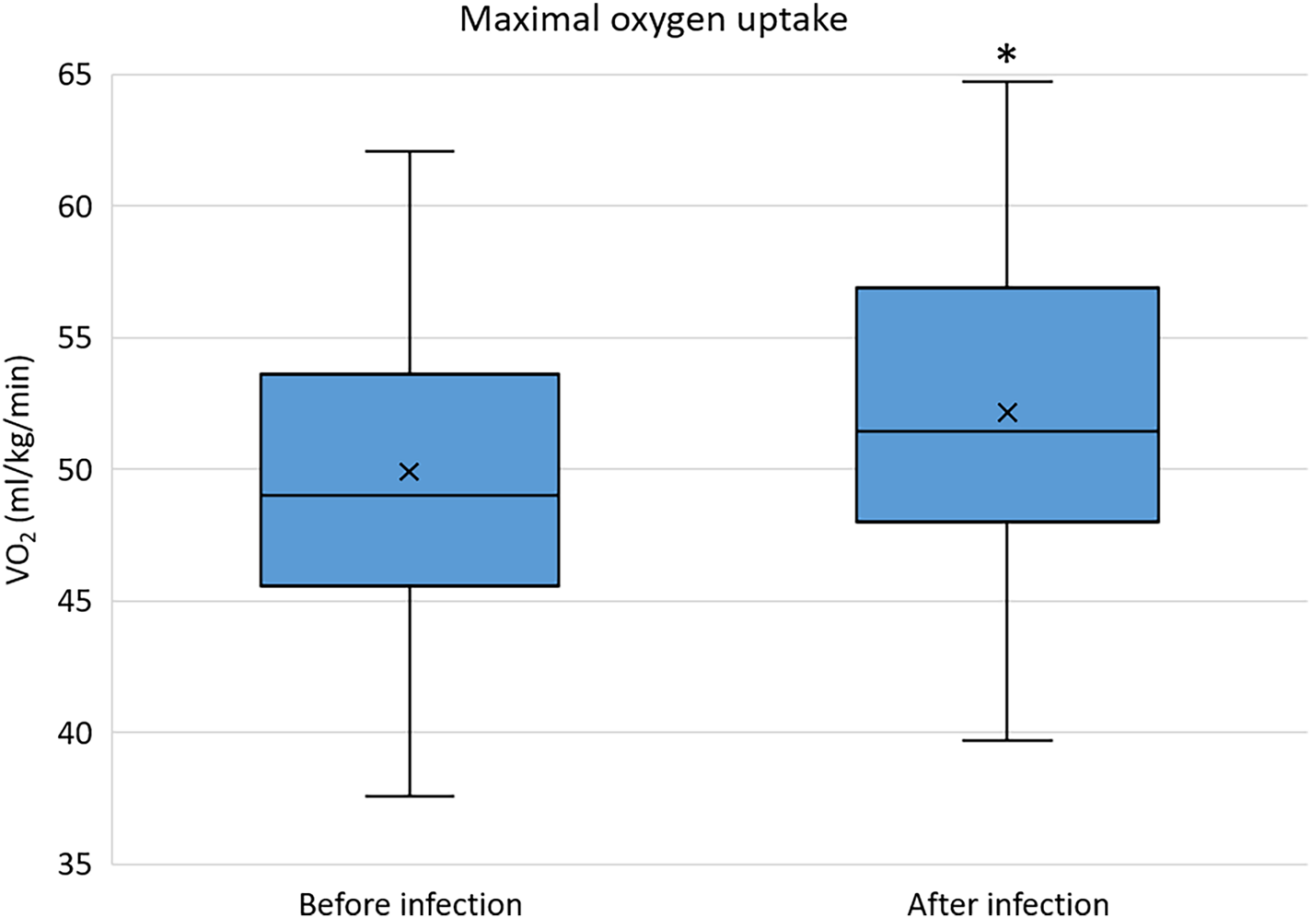
Maximal oxygen uptake of the examined asymptomatic elite athletes before and after the SARS-CoV-2 infection (n = 62). Abbreviations: VO2, oxygen uptake; *, *p* < 0.005.

**Figure 3:**
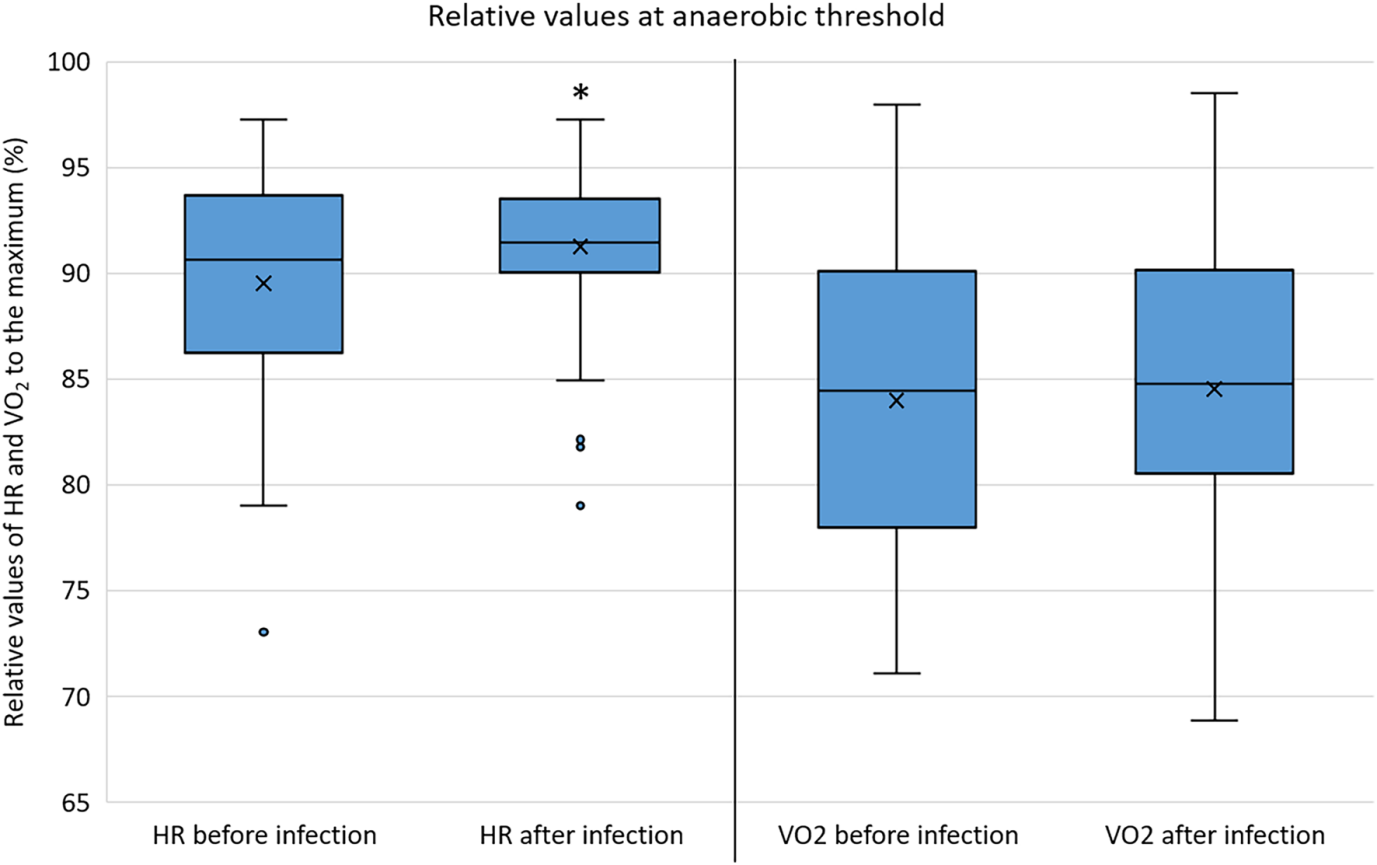
Relative heart rate and oxygen uptake at the anaerobic threshold in relation to the maximal values of the examined asymptomatic elite athletes before and after the SARS-CoV-2 infection. Abbreviations: HR, heart rate; VO2, oxygen uptake; *, *p* < 0.005.

In 62 athletes, previous CPET results were also available (Table 1.). Follow-up time between CPET examinations before and after the SARS-CoV-2 infection was 0.74 years (IQR: 0.61–1.78 years). The CPET exercise time proved to be longer after the infection compared to the previous results (pre- vs. post-infection: 13.0 min (IQR: 11.0–15.0 min) vs. 14.0 (IQR: 12.0–15.8) min, *p* = 0.003). In terms of V̇O_2max_ and ventilation, even higher values were observed on the CPET after the infection compared to the previous examinations. (Fig. 2) The athletes achieved similar maximal blood lactate levels during the exercise tests and spent a similar percentage at the anaerobic phase. At the anaerobic threshold, higher heart rate ratio to the maximal heart rate and similar oxygen uptake ratio to the V̇O_2max_ were measured. (Fig. 3) Compared to the previous results, a slight decrease of maximal heart rate was observed on the CPET after the infection (Table 1.), however, results corrected for age showed no significant change in maximal heart rate (adjusted pre- vs. post-infection: 190.6 ± 12.5 vs. 188.2 ± 12.0 BPM, *p* = 0.086). No significant differences were observed between VE/CO2 slopes before and after the infection. However, individual cases of decreased exercise capacity (more than 10% decrease of V̇O_2max_ at the post-COVID CPET compared to the previous examinations) were also confirmed by the CPET results (N = 6 [9.7%]).

Detailed evaluation revealed resting or exercise-induced atrial or ventricular arrhythmias or significant ST-T changes (ST-depression, T-wave inversion) in 8 (4.8%) athletes, while no pathological resting or exercise induced corrected QT interval changes (calculated by the Bazett formula) were found in any of the athletes^25^. In these cases, no direct connection between ECG abnormalities and the infection were proven, but further evaluation and close follow-up were recommended to exclude any potentially malignant arrhythmias or cardiac pathologies. (Table 2.) Behind the above arrhythmias, no structural cardiac abnormalities were revealed by the detailed cardiac evaluation. The exercise induced sustained ventricular tachycardia proved to be a Belhassen-type arrhythmia. Out of the ST-depression cases, one athlete (with descending ST depression and T-wave inversion in inferior leads during the CPET) had non-significant ischemic heart disease, while another one had a coronary artery bridge due to the results of cardiac CT examinations. By hypertensive exercise blood pressure measurements and ambulatory blood pressure monitoring results, new initiation of antihypertensive therapy was necessary in 7 cases. (Table 2).

**Table 2:** Clinical findings of basic, cardiopulmonary exercise testing and further examinations and treatments among 165 asymptomatic elite athletes 93.5 days (IQR: 66.8–130.0 days) after SARS-CoV-2 infection.

Clinical findings of asymptomatic elite athletes (N = 165)	N	%
**Laboratory test findings**		
Slightly increased high-Sensitive Troponin T	1	0.6
**Echocardiographic findings**		
Preserved left/right ventricular EF	1	0.6
Slightly increased pulmonary artery pressure	2	1.2
Diastolic dysfunction Grade I	1	0.6
Barlow type mitral valve + MAD	1	0.6
Left ventricular hypertrabecularization	2	1.2
**CPET findings**		
Exercise induced VPB	4	2.4
Exercise induced VT	1	0.6
Exercise induced SVPB	1	0.6
Exercise induced SV runs	1	0.6
Exercise induced STD	3	1.8
Decreased FEV1	1	0.6
**Holter ECG findings**		
Significant VPB %	1	0.6
SV runs	1	0.6
**Cardiac CT findings**		
Non-significant ischaemic heart disease	1	0.6
**Treatments**		
New antihypertensive therapy	7	4.2

*EF* Ejection fraction; *MAD* Mitral annular disjunction; *CPET* Cardiopulmonary exercise testing; *VPB* Ventricular premature beats; *VT* Ventricular tachycardia; *SVPB* Supraventricular premature beats; *SV* Supraventricular; *STD* ST-segment depression; *FEV1* Forced expiratory volume during the first second; *CT* Computer tomography.

In 22 (13.3%) asymptomatic elite athletes, just the echocardiography (n = 7, 4.2%) or the CPET examinations (n = 15, 9.1%) revealed cardiovascular pathologies requiring treatment or follow-up. In cases of cardiac pathologies, further examinations, restrictions in sports activity, and follow-up were recommended according to the current European guidelines^30^.

### Results of athletes with positive findings or ongoing symptoms during the second visit

The results of those elite and non-elite athletes who still had symptoms during the second visit or had positive clinical findings (n = 18, elite athlete: n = 9) were evaluated separately and are detailed below.

At the time of the control measurements, 11 athletes were still symptomatic (elite athletes: n = 5), although previously all of them had only mild symptoms in the acute phase of the infection. Symptoms were decreased exercise capacity (n = 4), palpitations (n = 3), exercise-induced shortness of breath (n = 2), worsening symptoms of asthma bronchiale (n = 2), or peripheral skin symptoms (n = 1). (Table 3).

**Table 3:** Clinical findings of basic, cardiopulmonary exercise testing and further examinations and treatments among 18 athletes with previous positive results or ongoing symptoms during the second visit.

Clinical findings of athletes with ongoing symptoms or previous positive results (N = 18)	N
**Ongoing symptoms**	
Decreased exercise capacity	4
Palpitations	3
Exercise induced dyspnoea	2
Worsening symptoms of asthma bronchiale	2
Peripheral skin symptoms	1
**Laboratory test findings**	
Slightly increased high-sensitive Troponin T	1
**CPET findings**	
Exercise induced VPB	3
Exercise induced nsVT	1
Exercise induced STD	1
**Holter ECG findings**	
Significant VPB %	2
Paroxysmal AF	1
**Cardiac CT findings**	
Anomalous coronary artery origin	1
Non-significant ischaemic heart disease	1
**Treatments**	
Right coronary artery PCI	1

*CPET* cardiopulmonary exercise testing; *VPB* ventricular premature beats; *nsVT* non-sustained ventricular tachycardia; *STD* ST-segment depression; *AF* atrial fibrillation; *CT* computer tomography; *PCI* percutaneous coronary intervention.

In an asymptomatic case, elevated hs Troponin T levels were measured repeatedly from the first step visit, and similar values were measured during a more than 6-month follow-up. During this time, no symptoms appeared, and all examinations—including cardiac MR—were negative. In this case, the hs Troponin changes were considered as an individual characteristic without cardiac diseases.

One athlete, who previously had mild acute symptoms due to COVID-19 disease for 12 days, suffered from a long-standing mild, stabbing chest pain starting almost 2 weeks after the onset of the first symptoms, and visited our Clinic for the first time 2 months after the starting symptoms of the disease. Due to these late symptoms, a cardiac MR examination was carried out and revealed preserved left and right ventricular ejection fractions, and infero-lateral and apical-lateral sub-epicardial late gadolinium enhancement without oedema as a potential sign of previous myocarditis. A follow-up cardiac MR carried out 8 months later detected the regression of these pathological signs (late gadolinium enhancement area 2020.11.: 9% vs. 2021.07.: 5%). Due to the timing of the infection and in the absence of other infections, this case was considered as a previous COVID-19 myocarditis.

**Figure 4:**
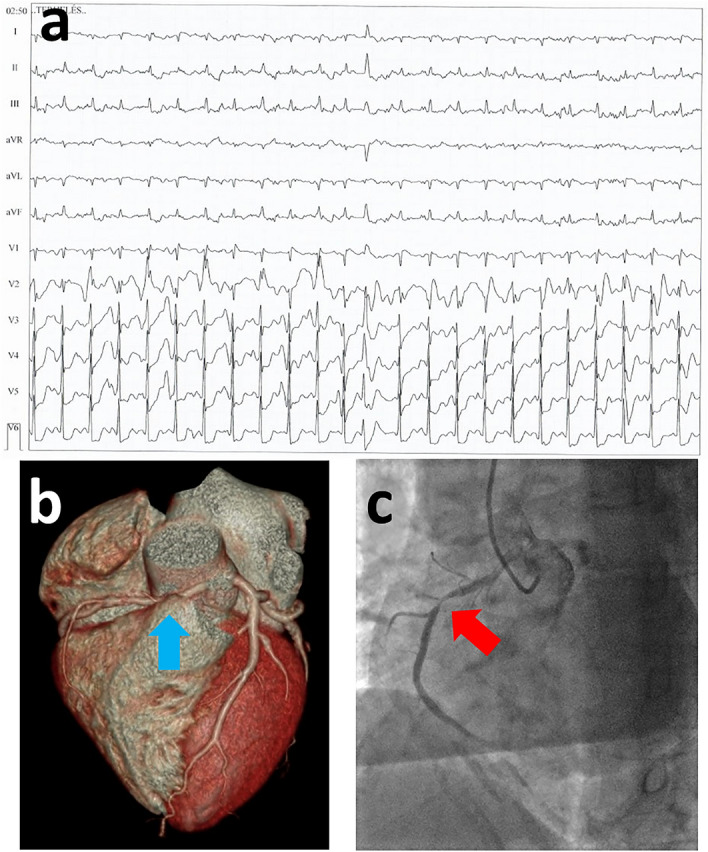
The case of a 60-year-old female amateur runner, with non-COVID-19-related findings. In a master female amateur runner with treated hypertension, the SARS-CoV-2 caused mild symptoms, and she still suffered from weakness 3 months after the infection. On her first visit, echocardiography revealed grade I-II. mitral insufficiency and the resting ECG showed incomplete right bundle branch block and horizontal 1 mm ST-depression in V4-V6 leads; no major alterations were found in her laboratory examination. During her second visit, he CPET examination revealed significant horizontal 2 mm ST-depression in V4-V6 (panel **A**). The patient was referred to cardiac CT, where the right coronary artery had the origin from the left aortic sinus of Valsalva and turned immediately rightwards in a very acute angle and traversed in between pulmonary trunk and aorta before returning to its normal course. Also, on the same artery after leaving the pulmonary trunk and aorta, a significant atherosclerosis was revealed (panel **B**). Due to these findings, a percutaneous coronary intervention was recommended, which verified the anomalous origin of the right coronary artery and showed the significant stenosis of the same artery (panel **C**). Therefore, a drug-eluting stent implantation was carried out. Due to the anomalous origin of the right coronary artery, a stress echocardiography was also performed after the intervention to examine cardiac function during exercise. No ischemic regions, or wall motion abnormality was detected. The asymptomatic patient was advised to perform light-to-moderate intensity sport activities.

A non-elite master athlete with horizontal ST-depression in V4-V6 precordial leads, proved to have anomalous right coronary artery origin and significant coronary artery disease. The right coronary artery originated from the left aortic sinus of Valsalva and turned immediately rightwards in a very acute angle and traversed in between the pulmonary trunk and the aorta before returning to its normal course (Fig. 4).

**Figure 5:**
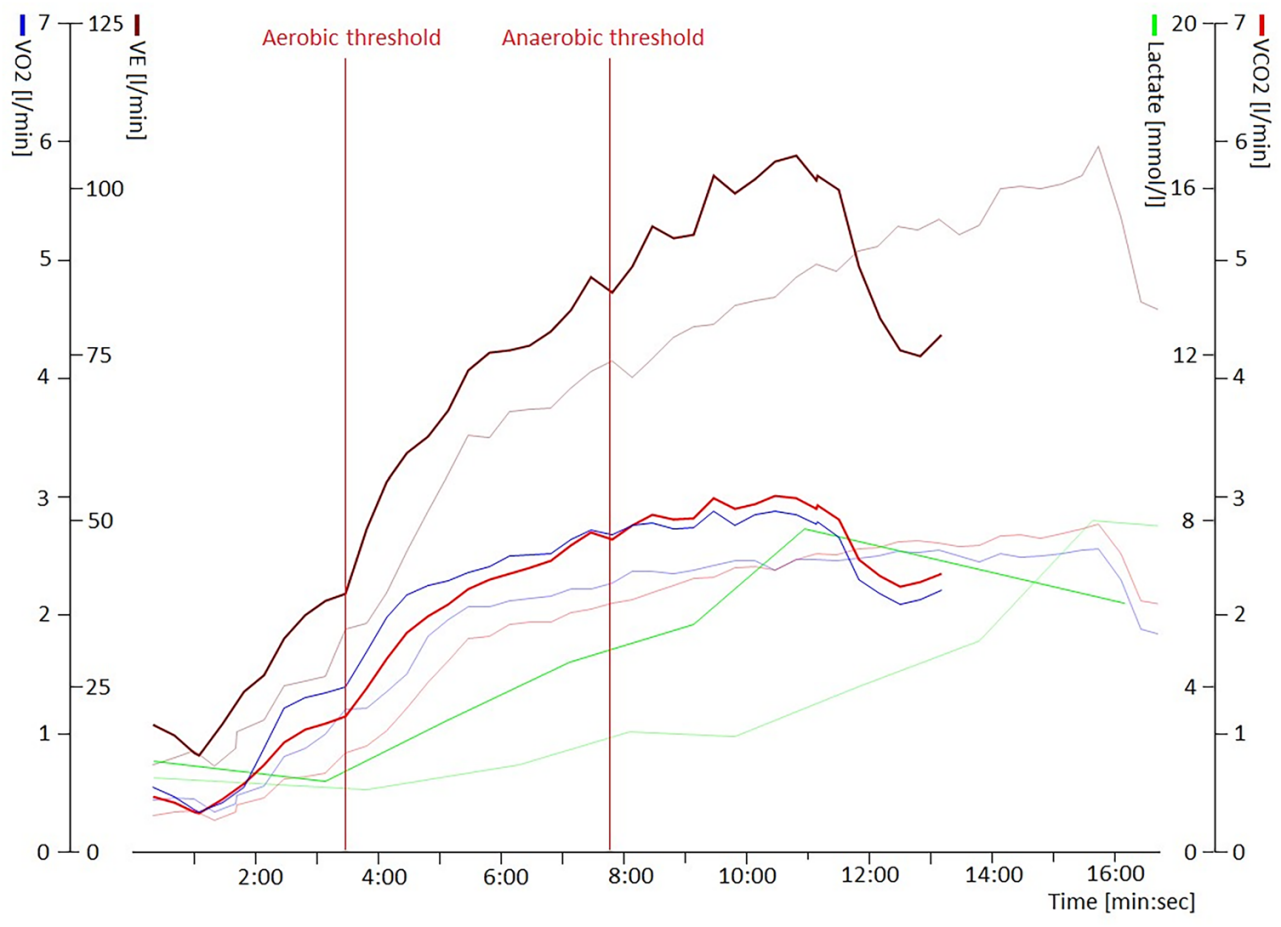
Decreased exercise capacity of a 19-year-old female water polo player after SARS-CoV-2 infection. On the graph, two CPET examinations are shown, between the two exanimations the follow-up time was 0.61 year. The earlier results of the examinations are shown with pale colours, the examinations after the SARS-CoV-2 are shown with sharp lines. The athlete achieved shorter running time on the same CPET protocol, with approximately the same ventilation (brown lines), slightly higher oxygen uptake (blue lines) and carbon dioxide production (red lines), worse metabolic adaptation to sports activity, which is represented by the increased lactate levels (green lines). The aerobic and anaerobic thresholds are represented with two vertical lines during the post-infection examination. In conclusion, multiple negative effects on her fitness status can be observed in this case. Further examinations revealed the worsening of her previously known asthma bronchiale symptoms and her treatment was optimized. Abbreviations: VO2, oxygen uptake; VE, ventilation; VCO2, carbon-dioxide production.

In an elite athlete, who still had effort dyspnea symptoms at the time of CPET examinations, decreased exercise capacity was observed. This athlete suffered of asthma bronchiale diagnosed before the SARS-CoV-2 infection and treated with optimal medical therapy. However, the symptoms of asthma bronchiale worsened in the long term following the infection. As a consequence, a significant decrease was measured in the fitness status comparing the CPET results before and after the infection. After pulmonary examinations, asthma bronchiale treatment was optimized and the symptoms of the athlete resolved (Fig. 5).

In case of a master athlete, who had palpitations and fatigue after a moderate symptomatic SARS-CoV-2 infection, multiple ventricular premature beats, ventricular couplets, a short non-sustained ventricular tachycardia (5 beats) and multiple supraventricular premature beats were recorded on the CPET. Multiple polymorphic ventricular couplets and a 19-beat-long paroxysmal atrial fibrillation were recorded on the 24-h Holter ECG. Due to the positive findings on the CPET and Holter ECG examinations and to the various cardiovascular risk factors, a cardiac CT was carried out. A borderline significant stenosis was revealed on the proximal part of the right coronary artery. Stress echocardiography was performed for further evaluation, but no ischaemic signs were revealed.

The pathological coronary artery diseases revealed in two amateur master endurance athletes (1 of them asymptomatic with ST-T abnormalities on the resting ECG and 1 with palpitations after SARS-CoV-2 infection) emphasize the importance of cardiology screening and early cardiology evaluation in this higher risk population.

In cases of symptoms or cardiac pathologies, further examinations, restrictions in sports activity, and follow-up were recommended according to the current European guidelines^30^.

## Discussion

Previous data are scarce about the cardiopulmonary status of athletes after returning to maximal intensity training following a SARS-CoV-2 infection. In our study, more than 3 months after SARS-CoV-2 infection, most of the examined asymptomatic elite athletes had satisfactory fitness levels. However, regarding the elite athletes, 2,8% (n = 5/174) were still complaining of COVID-19-related symptoms. The low percentage of symptomatic elite athletes in our study suggests no connection between training after the first negative examinations and long-standing symptoms. As the incidence of these long-standing post-Covid-19 symptoms decrease with the elapsed time after the breakout of the disease, the ratio of remaining symptoms was, not surprisingly, much higher (18%) in a study examining an elite athlete group less than 3 weeks after the infection^18^.

By examining 789 athletes, out of whom 58% had prior symptomatic COVID-19, Martinez et al. found that 0.6% had cardiac MRI findings suggesting inflammatory heart disease^15^. In addition to these findings, they revealed changes in troponin levels (0.8%), ECG (1.3%) and echocardiography (2.5%) 19 days after SARS-CoV-2 infection^15^. In our study, no new onset of inflammatory heart disease was revealed during the long-term follow-up period. However, a supposedly SARS-CoV-2-related previous myocarditis was revealed in a long-term symptomatic elite athlete. In two athletes, elevated hs Troponin T levels proved to be individual or sports-related after thorough investigation. These results are concordant with the work of Vágó et al., who did not find acute cardiac involvement among 12 young elite athletes shortly after the infection^7^. All together, our results show that cardiac involvement after asymptomatic or mildly symptomatic SARS-CoV-2 infection in athletes is rare. These observations are in concordance with the results of a larger cohort study by Moulson et al. as well with the results of 147 highly trained athletes of Szabo et al.^33,34^ Systematic reviews summarizing data of long-term cardiac imaging manifestations also found low risk for myocardial or pericardial involvement after a SARS-CoV-2 infection^35,36^.

Our study was carried out on a mixed group of athletes most of whom were asymptomatic or had mild-to-moderate symptoms during the COVID-19 infection. Referring to previous literature data and also our previous results, average V̇O_2max_ of the examined mainly mixed team elite asymptomatic athlete population proved to be satisfactory 3 months after the infection^37^. In a subgroup of asymptomatic elite athletes who also had previous CPET data from before the SARS-CoV-2 infection, except for a few cases, no significant decrease in V̇O_2max_ could be observed, but a significant increase in mean exercise time, V̇O_2max_, ventilation, and heart rate at anaerobic threshold could be measured. These results could be explained with the fast recovery of the athletes as well as the intensive trainings before world competitions like the Olympic Games held in 2021. Different training phases at the time of the CPET measurements before and after the SARS-CoV-2 infection could also affect these results. The data of Cavigli et al. support our data, as they did not find deviations on the resting spirometry in 90 asymptomatic or mildly symptomatic young athletes 30 days after recovering from a SARS-CoV-2 infection and compared to a healthy athlete group^12^. Moreover, the authors did not find limitations of cardiac or pulmonary functions during CPET shortly after the infection^12^. Komici et al. also did not find a decrease in CPET parameters after COVID-19 in a short-term follow-up after the infection, they only found decrease in the forced expiratory volume in the first second (FEV1)^20^. In our study, no changes in FEV1 or VE/VCO_2_ slope were measured due to the COVID-19 infection in asymptomatic elite athletes. Cavigli et al. found similar VE/VCO2 slope results in athletes after suffering a SARS-CoV-2 infection^12^.

In a football team cohort study of 30 athletes with (n = 18) and without (n = 12) previous SARS-CoV-2 infections were compared to each other and to their own previous values via spirometry and exercise stress ECG–performed 15 days after complete recovery. Compared to the previous personal measurements, a significant decrease was found in the SARS-CoV-2-infected group, however the SARS-CoV-2-negative athletes also suffered the same amount of detraining. Not surprisingly, these data suggested that COVID-19 infection could cause a significant decrease in fitness around 1 month after the onset of the disease, which exceeded the predicted decrease from detraining^38^. Unfortunately, CPET values for this group are not available. In contrast, in our athlete group no significant decrease, but increase was found in some parameters of the CPET, which could be explained by the longer follow-up time after the infection, thus more time was allowed for rehabilitation and achieving maximal intensity trainings. Csulak et al., who examined 46 professional swimmers out of whom 14 were SARS-CoV-2-infected, did not find major differences in CPET results before and after the infection^39^.

Among 16 male elite volleyball players after mild SARS-CoV-2 infection with 22 days of training cessation followed by 20 days of training before the CPET, a Serbian workgroup found good aerobic fitness levels (VO_2max_: 44.1 ± 3.4 ml/kg/min; VE_max_: 152.4 ± 18.7 l/min; HR_max_: 183.0 ± 8.3 BPM)^40^. They indicated that the infection had no effects on any athletes, which is generally true about our study group as well, with a few exceptions, where a decrease could be observed in the fitness values^40^. Among these volleyball players the authors found that, the decreases in fitness status exceeded the expected decrease due to the previous detraining period.

The detailed screening and follow-up could help to detect alterations, supposedly SARS-CoV-2-related and unrelated ones as well. Extending the routine screening with echocardiography and CPET examination in elite asymptomatic athletes revealed additional cardiovascular pathologies in 22 (13.3%) athletes, thereby we advise to perform these examinations preferably regularly, but at least after infections (e.g. SARS-CoV-2 infection) in all elite athletes to reduce the risk of sudden cardiac death. Although no direct connection with the infection could be proven, detailed evaluation revealed resting or exercise-induced atrial or ventricular arrhythmias or hypertension in some athletes. Although no structural abnormality was revealed in the back, these athletes need special attention and close follow-up. During the examinations, some non-COVID-19-related severe alterations were also revealed, such anomalous coronary artery origin and ischemic heart disease. Since these diseases are not connected to COVID-19, these should be considered as side-findings. However, these findings highlight the importance of the widely implemented extended cardiology examinations of athletes after SARS-CoV-2 infection as a worldwide athletic screening never seen before. Furthermore, it would be more favourable if all elite athletes would undergo a detailed cardiology screening at least once in a lifetime to reveal cardiovascular diseases and to reduce risk of sudden cardiovascular death.

### Limitations

This is a single center study carried out solely among Hungarian athletes. The examined population should be broadened and divided to the different variants of the SARS-CoV-2 virus. The pre-SARS-CoV-2 infection CPET data were achievable retrospectively and at the time of those measurements the athletes were in different training phase. It would be more ideal to perform the CPET examinations at the same training phase. A long-term follow-up should also be performed to evaluate the even longer impact of the SARS-CoV-2 infection on the athletes’ performance and fitness status, however the results 3 month after the infection suggest no decrease in the performance in most athletes.

## Conclusions

More than 3 months after SARS-CoV-2 infection, most of the athletes had satisfactory fitness levels, and intensive sport activity proved to be safe in most of the cases. By the CPET results, also improvement was found comparing before and after SARS-CoV-2 infection results in the V̇O_2max_ and maximal ventilation. However, some patients having symptoms or positive clinical findings such as arrhythmias required further examinations and follow-up. The percentage of long-symptomatic elite athletes was low, and the percentage of positive findings related SARS-CoV-2 infection was low as well. Moreover, due to the detailed screening, some significant diseases independent of SARS-CoV-2 infection were revealed.

Overall, asymptomatic athletes can continue their trainings after COVID-19 infection following appropriate cardiovascular examinations safely. With well-built-up training-plans, good exercise fitness could be achieved in most of the athletes in three months, and the infection did not restrict their sports career in the long term.

## Data Availability

The datasets used and analysed during the current study are available from the corresponding author on reasonable request.
